# Supplementary material for: Post-stroke changes in brain structure and function can both influence acute upper limb function and subsequent recovery
Source: Neuroimage Clin. 2025 Feb 13;45:103754. doi: 10.1016/j.nicl.2025.103754 (PMC11889610; doi:10.1016/j.nicl.2025.103754)
Supplement: Supplementary Data 1 [file mmc1.pdf]

Supplemental Information

Post-stroke changes in brain structure and function can both influence acute upper limb function and subsequent recovery

Catharina Zich, Nick S Ward, Nina Forss, Sven Bestmann, Andrew J Quinn, Eeva Karhunen, Kristina Laaksonen

Supplemental Information – Table 1: Clinical details of the patients.

Study	Patient	Gender	Age	AH	Site	NHPT	
						T_0	T_1
Laaksonen et al., 2012	1	M	60	R	C	120	101
	2	F	67	L	S	38	30
	3	F	72	L	C	33	24
	4	F	55	R	CS	120	120
	5	M	68	L	CS	58	34
	6	F	84	R	C	120	64
	7	F	68	L	S	120	120
	8	F	72	R	CS	120	120
	9	M	62	L	CS	47	43
	10	F	74	R	S	120	44
	11	M	78	L	S	120	30
Parkkonen et al., 2018	1	F	59	R	S	180	180
	2	M	59	R	C	77	28
	3	M	57	L	C	180	55
	4	M	68	R	S	28	28
	5	M	71	R	CS	35	26
	6	F	59	L	C	35	22
	7	M	76	L	CS	29	23
	8	M	74	L	S	39	31
	9	M	66	R	CS	41	28
	10	F	68	R	C	32	21
	11	F	59	R	S	37	26
	12	M	45	R	CS	21	20
	13	F	58	R	CS	23	23
	14	F	66	L	C	180	180
	15	F	73	L	CS	180	131
	16	F	67	R	CS	42	28
	17	M	75	R	CS	180	180
	18	F	75	L	S	180	180
	19	M	64	L	S	180	180
	20	M	65	L	S	180	32
	21	F	74	R	S	180	180
	22	M	67	R	CS	180	180
	23	M	47	R	CS	180	180
	24	F	78	R	CS	180	180
	25	M	66	R	S	155	20

AH, affected hemisphere; R, Right; L, Left; C, cortical; CS, cortico-subcortical; S, subcortical; T0, 1–7 days; T1, 1 month

Supplemental Information - Figures

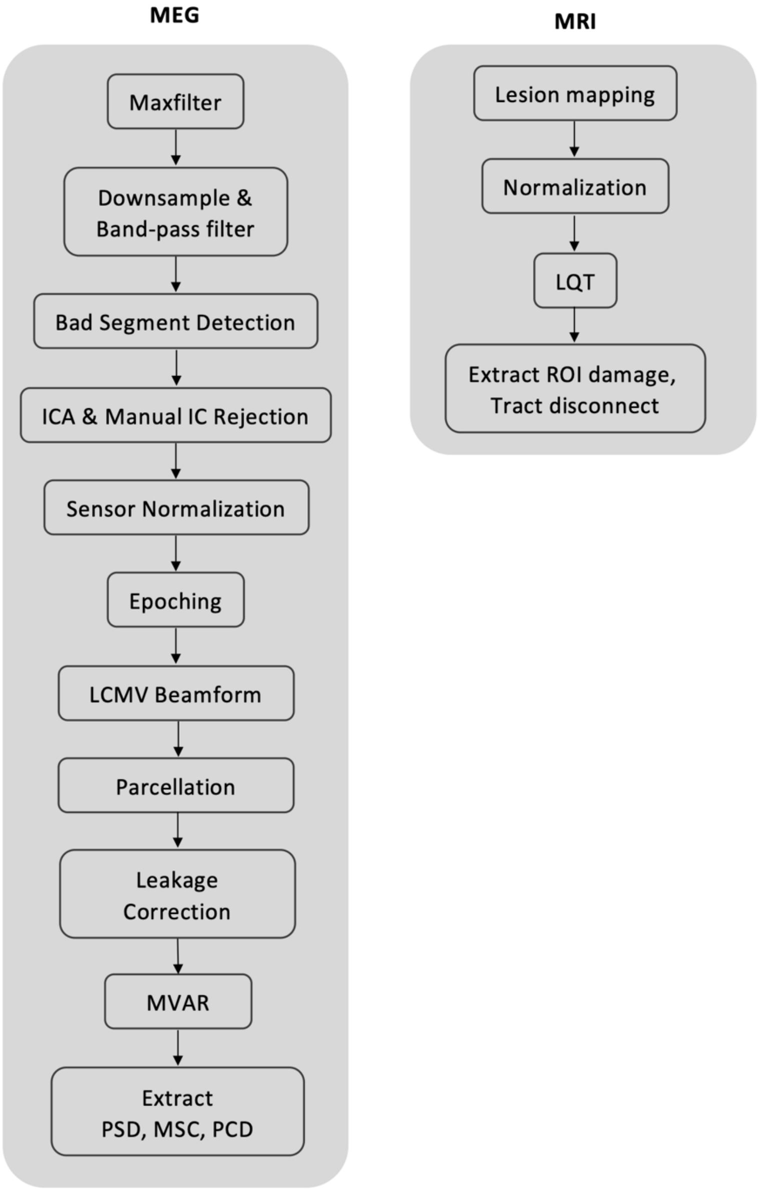

SI Fig. 1. *A schematic for the processing pipeline.*

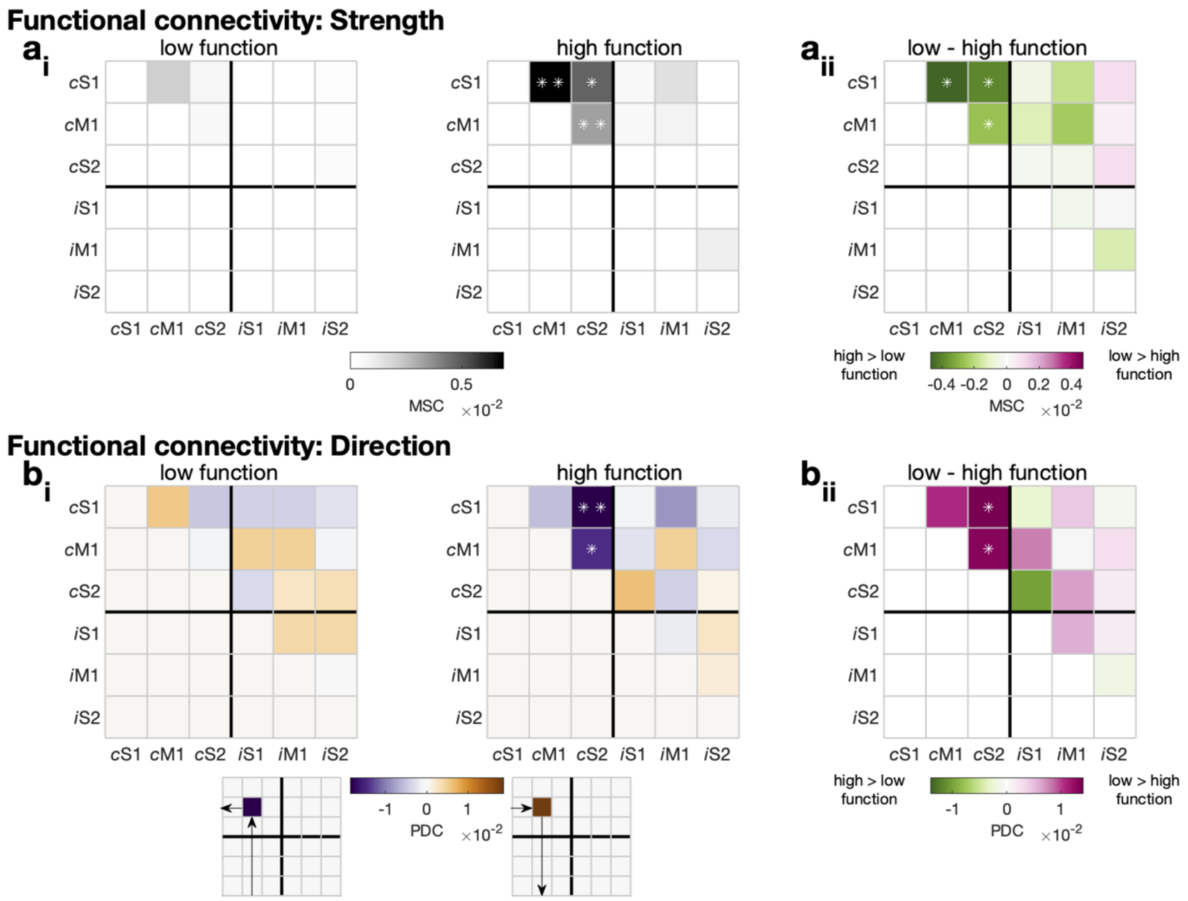

SI Fig. 2. *Connectivity strength and direction for low function (left) and high function (right) patients.*
*ai) Connectivity strength for low function (left) and high function (right) patients. Connections, where the strength differs significantly from zero are highlighted (p<0.05 *, p<0.01**).*
*aii) Difference in connectivity strength between low and high function patients. Significant cells are highlighted (p<0.05 *, p<0.01**).*
*bi) Connectivity direction for low function (left) and high function (right) patients. The direction for purple cells is row to column, whereby the direction for brown cells is column to row (see also the legend below). Connections, where the direction differs significantly from zero, are highlighted (p<0.05 *, p<0.01**).*
*bii) Difference in connectivity direction between low and high function patients. Significant cells are highlighted (p<0.05 *, p<0.01**).*

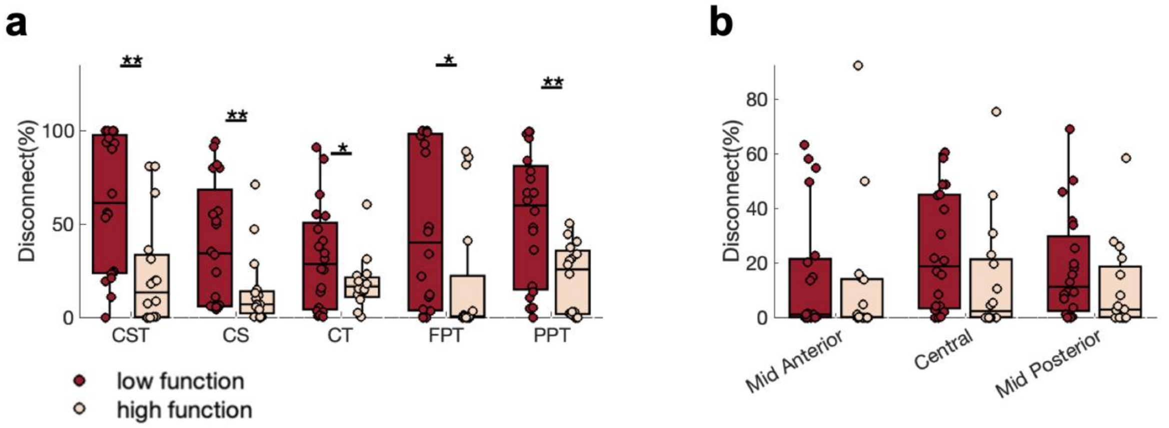

SI Fig. 3. *Tract disconnect for low and high function patients.*
*a) Motor projection connections (corticospinal tract [CST], corticostriatal pathway [CS], corticothalamic pathway [CT], frontopontine tract [FPT], parietopontine tract [PPT]). Significant differences between groups are highlighted (p<0.05 *, p<0.01**).*
*b) Motor commissural connections (mid-anterior corpus callosum, central corpus callosum, mid-posterior corpus callosum). Significant differences between groups are highlighted (p<0.05 *, p<0.01**).*

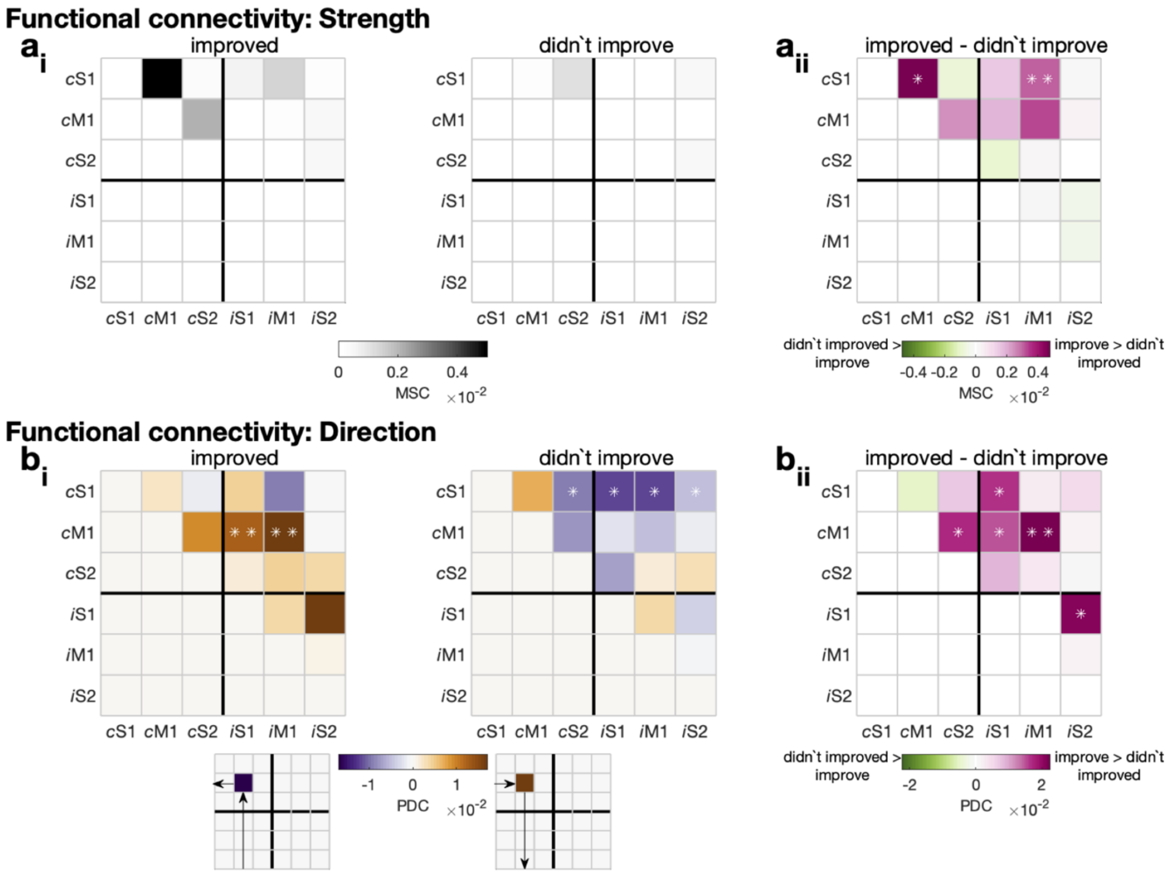

SI Fig. 4. *Connectivity strength and direction patients who improved (left) and patients who didn't improve (right).*
*ai) Connectivity strength for patients who improved (left) and patients who didn't improve (right). Connections, where the strength differs significantly from zero are highlighted (p<0.05 *, p<0.01**).*
*aii) Difference in connectivity strength between patients who improved and patients who didn't improve. Significant cells are highlighted (p<0.05 *, p<0.01**).*
*bi) Connectivity direction for patients who improved (left) and patients who didn't improve (right) patients. The direction for purple cells is row to column, whereby the direction for brown cells is column to row (see also the legend below). Connections, where the direction differs significantly from zero, are highlighted (p<0.05 *, p<0.01**).*
*bii) Difference in connectivity direction between patients who improved and patients who didn't improve. Significant cells are highlighted (p<0.05 *, p<0.01**).*

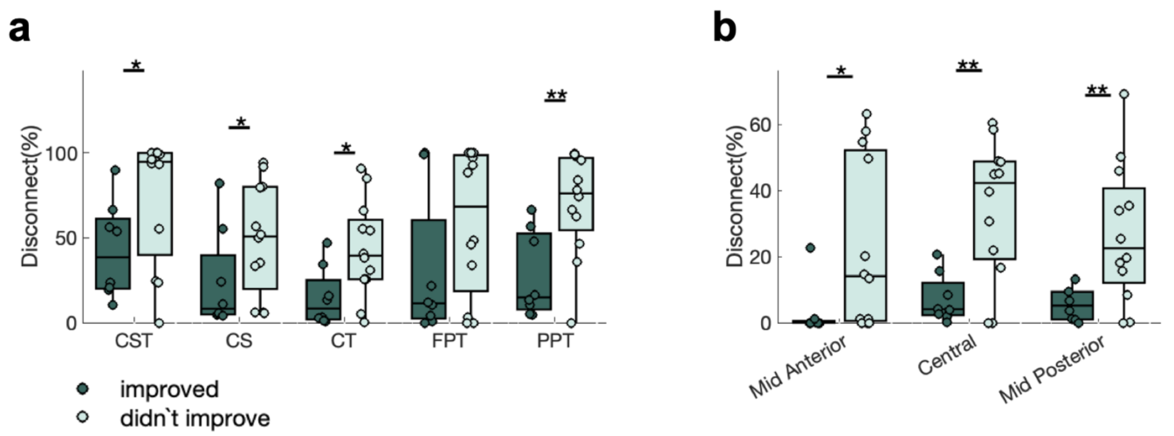

SI Fig. 5. *Tract disconnect metrics for patients who improved and patients who didn't improve.*
*a) Motor projection connections (corticospinal tract [CST], corticostriatal pathway [CS], corticothalamic pathway [CT], frontopontine tract [FPT], parietopontine tract [PPT]). Significant differences between groups are highlighted (p<0.05 *, p<0.01**).*
*b) Motor commissural connections (mid-anterior corpus callosum, central corpus callosum, mid-posterior corpus callosum). Significant differences between groups are highlighted (p<0.05 *, p<0.01**).*

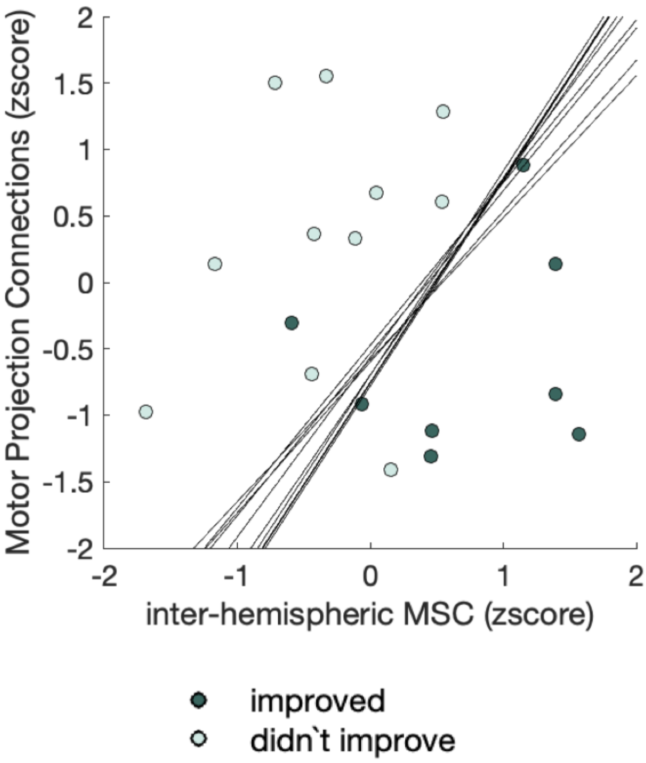

SI Fig. 6. *Predictors included in the final logistic regression model. Each line represents the best separating hyperplane for the classifier for each fold of the leave one out cross validation.*

Supplemental Information – Results

Recovery-related differences in functional brain connectivity direction (Partial directed coherence, PDC)

Group	From	To	df	t	p
**improved**	contralateral [ipsilesional] M1	ipsilateral [contralesional] M1	7	3.97	<0.01
	contralateral [ipsilesional] M1	ipsilateral [contralesional] S1	7	3.33	<0.01
**didn't improve**	contralateral [ipsilesional] S2	contralateral [ipsilesional] S1	11	-1.95	<0.05
	ipsilateral [contralesional] M1	contralateral [ipsilesional] S1	11	-1.93	<0.05
	ipsilateral [contralesional] S1	contralateral [ipsilesional] S1	11	-2.50	<0.05
	ipsilateral [contralesional] S2	contralateral [ipsilesional] S1	11	-2.62	<0.01
**improved > didn't' improve**	contralateral [ipsilesional] M1	contralateral [ipsilesional] S2	18	1.97	<0.05
	contralateral [ipsilesional] M1	ipsilateral [contralesional] M1	18	3.13	<0.01
	contralateral [ipsilesional] M1	ipsilateral [contralesional] S1	18	2.10	<0.05
	contralateral [ipsilesional] S1	ipsilateral [contralesional] S1	18	2.58	<0.05
	ipsilateral [contralesional] S1	ipsilateral [contralesional] S2	18	2.08	<0.05

Supplemental Information – Results

Model fit measures for the winning and two alternative models.

Inter-hemispheric MSC & Motor Projection Connections

Model Fit Measures

							Overall Model Test		
Model	Deviance	AIC	BIC	R^2_{McF}	R^2_{CS}	R^2_N	χ^2	df	p
1	13.3	19.3	22.2	0.508	0.495	0.669	13.7	2	0.001

CST

Model Fit Measures

							Overall Model Test		
Model	Deviance	AIC	BIC	R^2_{McF}	R^2_{CS}	R^2_N	χ^2	df	p
1	23.1	27.1	29.1	0.141	0.173	0.234	3.81	1	0.051

M1 and S1 β rebound

Model Fit Measures

							Overall Model Test		
Model	Deviance	AIC	BIC	R^2_{McF}	R^2_{CS}	R^2_N	χ^2	df	p
1	18.5	22.5	24.5	0.312	0.343	0.463	8.39	1	0.004

AIC Akaike information criterion
BIC Bayesian information criterion
R^2_{MCF} McFadden's R^2
R^2_{CS} Cox & Snell's R^2
R^2_N Nagelkerke's R^2
X^2 Chi-square
